# Professor Simpson

**Published:** 1854-12

**Authors:** 


Professor Simpson propounds the theory that Phlebitis is contagious, and, like epidemic erysipelas and puerperal fever, may be communicated by the hands or instruments of the operator. Hospital gangrene he would place in the same category. If this theory be true, a new corps of surgeons should be enlisted for the New York Hospital at this present writing, for those on duty are making sad havoc by spreading the poison, if the reported fatality by the latter disease be correctly stated.—*N. Y. Med. Gaz.*

